# Concentration Study of High Sensitive C - reactive Protein and some Serum Trace Elements in Patients with Benign and Malignant Breast Tumor

**Published:** 2015-10-01

**Authors:** Alireza Abdollahi, Abbas Ali-Bakhshi, Zahra Farahani

**Affiliations:** 1 Associate Professor of Pathology, School of Medicine, Imam Khomeini Hospital Complex, Tehran University of Medical sciences. Tehran, Iran; 2 Assistant Professor of Surgery, Imam Khomeini Hospital Complex, Vali-Asr Hospital, Surgery Ward, Tehran University Medical of sciences Tehran, Iran.; 3 Physiologists, Maternal Fetal and Neonatal Research Center, Tehran University Medical of Sciences, Tehran, Iran

**Keywords:** Benign and Malignant breast tumor, hs-CRP, Trace element

## Abstract

**Background** **:** Breast cancer is the most common invasive cancer in females worldwide. It accounts for 16% of all female cancers and 22.9% of invasive cancers in women. 18.2% of all cancer deaths worldwide including both males and females are from breast cancer. In this study we compared few serum elements in patients with benign and malignant breast tumor to find any related prognostic and predictive value.

**Subjects and Methods:** A case-control study was carried out in a hospital (Tehran - Iran) in 2012. Target population was divided in 2 groups; subjects with benign and malignant breast tumors. We did preoperative hematological test. Five milliliter fasting blood vein was collected, centrifuged in 3000 g for 15 minutes to obtain serum. We measured serum Calcium (Ca), Phosphorus (P), Magnesium (Mg), Zinc (Zn), and high sensitive-CRP, analyzed statistically and compared recorded elements in 2 groups by software package SPSS version 16. The level of significant was considered P < 0.05.

**Results:** Of 87 women, 49 cases with benign breast disease (group A) and 38 cases with breast cancer (group B) entered our study. Serum concentration of Ca, mg, and P in group A were higher than group B, however these differences were not significant. We found no significant correlation between serum Zn and type of tumor in our patients. On the other hand, a significant elevation in hs-CRP in patient with breast cancer was seen (P Value=.000).

**Conclusion** **:** Our results have shown similar concentration of Ca, Mg, Zn, P and completely different hs-CRP concentration in patients with benign and malignant breast disease.

## Introduction

 Breast cancer is the leading cause of cancer related death in women worldwide. In the United States, breast cancer is the most frequent female cancer, the second most common cause of cancer death in women after lung cancer and represents the main cause of death in women ages 20 to 59 years.^1^^,^^2^ Exposure to some carcinogens and trace elements play crucial roles in the development of variety of cancers. Elements and electrolytes with enzymatic cofactor properties, competitive role for binding positions, modifications in the permeability of cellular membranes, catalytic and angiogenesis function can involve in carcinogenic process. Elevated concentrations of zinc, Ca, Fe, Cu, P and Se have been cited in cancerous tissue.^3^^,^^4^^,^^5^ An investigation revealed that elevation of Fe, Cu, and Zn may have a promising effect in the initiation or progress of carcinogenic pattern. Excess accumulation of iron may consider as a risk factor of breast cancer. Concentration of Fe in malignant breast tissue samples and benign tissues samples was comparable (229  ± 121 vs. 49.1  ±  11.4 mg/L).^6^

In addition haemostatic factors have important role in inflammatory process and angiogenesis which interact with malignancy. Laboratory evidences show activation of coagulation and fibrinolytic systems in patients' diagnosed cancer. A study by Yigit et al. indicated higher plasma level of factor VΙΙΙ, ΙX, vWF and CRP in patients with breast tumor in compare to control group.^7^ High Sensitive – C Reactive Protein also is a sensitive marker of inflammation associated with cardiovascular diseases and cancers.^8^ Renee et al. and Min et al. showed elevated level of hs-CRP in prostate and gastric cancer patients.^9^^,^^10^

Breast cancer is one of common cancer between women in our country. In this study we compared few serum elements (Calcium (Ca), Phosphorus (P), Magnesium (Mg), Zinc (Zn) and high sensitive-CRP) in patients with benign and malignant breast tumor to find any related prognostic and predictive value**.**

## SUBJECTS AND METHODS

 This case-control study was carried out in surgery Ward and Central Laboratory of Imam Khomeini Hospital Complex in 2013. The inclusion criteria were females with a palpable mass in their breast or a lesion detected by mammography. We excluded patients with any other inflammatory and bone disease, taking any medications or who refused more work up such as needle biopsy or surgery in our medical center. A detailed history and physical examination of all participants were recorded. We divided our target population in 2 groups (49 cases with benign breast mass as group A and 38 cases with breast cancer as group B). Both groups were matched with respect to age and Where possible socioeconomic status. 

Benign and malignancy status was confirmed based on sono- mammographic finding, fine needle biopsy, and pathological results (gold standard) 

after tumor removal by surgery. We did preoperative hematological test. Five milliliter fasting blood vein was collected, centrifuged in 3000 g for 15 minutes to obtain serum, then measured and analyzed serum Ca, P, Mg, Zn and hs-CRP by kits (Bio system, Spain) and Auto analyzer (BT-3500, Italy). The assay was performed according to the manufacturer's instruction. We considered normal values for each analyt according to kit brochures. Finally we analyzed statistically and compared recorded elements in 2 groups by software package SPSS version 16; frequency by mean + SD and variables correlation with chi-square and Independent t- test were applied. The level of significant was considered P < 0.05.

All participants gave informed consent before entering the study. Patients’ data were considered secret, no extra cost was constrained and no intervention was performed in our study. The study design, protocols, procedures and informed consent form were approved by the Medical Ethic Committee of Tehran University of Medical Sciences (88-03-30-8920).

## Results

 Among 87 women, 49 cases with benign breast disease (group A) and 38 cases with breast cancer (group B) entered our study. The mean age of patients in group A was 43.75 ± 12.31 years and the mean age of patients in group B was 47.28 ± 15.87 years. In group A, 22 cases had a mass in right breast, 21 in left and 2 cases had bilateral breast mass while side of breast mass in counterpart group were 13, 22 and 4, respectively (Table 1). Serum concentration of Ca, Mg, and P in group A was higher than group B; however, these differences were not significant. We found no significant correlation between serum Zn and type of tumor in our patients. On the other hand, a significant elevation in hs-CRP in patient with breast cancer was seen (P Value=.000). The comparison between variables concentrations in both groups was shown in Table 2.

## Discussion

 Breast cancer rates are much higher in developed nations compared to developing ones.

**Table 1:** characteristic of two patient groups: A: Benign Breast mass, B: Breast cancer

**Total of patients = 87**			
**Group A (Benign Breast mass )**			
	** Mean age = 43.75 ± 12.31 years NO: 49 Cases**		
	**Side of mass**	** 22 cases in right breast**	
		**21 cases in left breast**	
		**2 cases in had bilateral mass**	
**Group A (Breast cancer )**			
	**Mean age = 47.28 ± 15.87 years NO: 38 Cases**		
	**Side of mass**		**13 cases in right breast**
			**22 cases in left breast**
			**4 cases in had bilateral mass**

There are several reasons for this, with possibly life-expectancy being one of the key factors. Breast cancer is more common in elderly women; women in the richest countries live much longer than those in the poorest nations. The different lifestyles and eating habits of females in rich and poor countries are also contributory factors, experts believe. Breast cancer is the most common invasive cancer in females' worldwide.^5^^-^^7^ It accounts for 16% of all female cancers and 22.9% of invasive cancers in women. 18.2% of all cancer deaths worldwide, including both males and females, are from breast cancer.^8^ According to the National Cancer Institute, 232,340 female breast cancers and 2,240 male breast cancers are reported in the USA each year, as well as about 39,620 deaths caused by the disease. Breast cancer is the second most common newly diagnosed cancer and second leading cause of cancer death among women in the US. Breast cancer (women only) was the second most common cancer with nearly 1.7 million new cases in 2012.^6^

It seems changes in the levels of certain trace elements can increase oxidative stress resulting breast cancer.^11^^,^^12^

Although many studies have shown the modulation of blood trace elements as diagnostic markers of the disease process and its possible relationship etiologically in the malignant breast tumor,^13^^,^^14^ according to our results, these serum elements disparities were not noticeable in benign and malignant groups.

The relation between serum Ca and breast cancer risk remains controversial. An inverse relation between dietary calcium intake and breast cancer risk was reported due to anti-proliferative and pro-differentiation effects on mammary cells.^13^^,^^15^ however, we saw no significant evidence for different serum calcium levels in patients with breast cancer in compare to the other group. We thought that reliance on a single serum calcium measure in women with different age and BMI may affect on our results. Sprague et al. also had found no correlation between either total or ionized serum calcium and breast cancer risk.^15^ In contrast to our finding Siddiqui. et al. have reported a higher Ca level in blood of malignant cases than in those of their benign counterparts.^14^

There are several reports that showed increased amount of phosphorus in the blood as indicative for the existence of unidentified cancerous tumor^16^ but based on our results both groups had normal blood phosphorus concentration. De Jorge et al. also reported that the mean value inorganic phosphorus in the blood serum of 23 women with carcinoma was in the normal range.^17^

Serum magnesium deficiency (Mg) has a notable role in cell proliferation, DNA mutations resulting carcinogenesis changes but the role of Mg in tumor development and in tumor pathology is intricate.^13^ we found no difference in 2 groups' Mg concentration which our results compatible with Seltzer et al. report. Nine of 10 patients with breast cancer had serum magnesium concentrations within normal limits.^18^

**Table 2:** Comparison of serum factors concentration in two groups

**Variables**	**Group A** * **N=49**	**Group B** ** **N=38**	**P value**
**Ca (mg/dl) (mean+ SD)**	9.0429 + .72	8.7881 + .90	.140
**P (mg/dl)** **(mean+ SD)**	4.0082 + 1.26	3.9476 + 1.05	.807
**Mg (mg/dl)** **(mean+ SD)**	2.0418 + .37	1.9357 + .32	.159
**Zn (mg/dl)** **(mean+ SD)**	68.9592 + 18.49	69.3571 + 14.23	.910
**hs-CRP (mg/L)** **(mean+ SD)**	3.6365 + 3.02	9.1095 + 4.59	.000

* Group A; Patient with benign tumor

** Group B; Patient with malignant tumor

Present study inconsistent to another study indicated that the serum concentrations of zinc in breast cancer patient remained unaltered.^12^ On the other hand Pasha et al. depicted that Average plasma concentrations of Zn was significantly higher in cancer patients compared with controls.^19^

We saw in this study, hs-CRP level in breast cancer group was notable. Many tumors arise from a chronic inflammatory site or they trigger an inflammatory cascade resulting formation of inflammatory factors and CRP elevation.^20^ Some reported papers hypothesized that elevated highly sensitive CRP in poor prognosis breast cancer cases may be related with age, BMI, malignancy stage and inflammatory response to tumor. ^21^ Allin et al. also revealed that preoperative hs-CRP level may provide short-term prognostic information as a subclinical marker of tumor stage, grade, size and presence of metastatic disease.^20^

We did not consider patients' BMI, menopausal status or pregnancy which all these factors could be efficient. Although we tried to do adjust for some potential confounders, other confounding factor like physical activity was ignored.

For research progress in future investigation we suggest focus on other metal and elements like Fe, Cu, Se and consideration of calcium with related factors such as parathyroid hormone and vitamin D. Applying advanced measurement methods and devices such as Atomic absorption or using multi centers data may affect on the results.

## CONCLUSION

 In conclusion, we have shown that correlation between blood trace elements and breast malignancy may be related to other factors rather than type of tumor but positive relation between hs-CRP and malignant breast tumor may provide a prognostic and predictive value for hs-CRP measurement. 

The restrictions in this study include the low number of patients and single-center status of the survey. Therefore, a study with more samples and with multi-center status is recommended.
